# Fasting plasma glucose level-based formula for estimating starting daily dose in basal-bolus insulin therapy

**DOI:** 10.1038/s41598-023-28138-6

**Published:** 2023-01-19

**Authors:** Mototsugu Nagao, Taro Harada, Kyoko Tanimura-Inagaki, Shunsuke Kobayashi, Izumi Fukuda, Hitoshi Sugihara, Shinichi Oikawa

**Affiliations:** 1 Department of Endocrinology, Metabolism and Nephrology, Graduate School of Medicine, Nippon Medical School, Tokyo, Japan; 2 Diabetes and Lifestyle-Related Disease Center, Fukujuji Hospital, Japan Anti-Tuberculosis Association, 3-1-24 Matsuyama, Kiyose, Tokyo 204-8522 Japan

**Keywords:** Type 2 diabetes, Hormonal therapies

## Abstract

There is no standard formula for estimating the starting daily dose (SDD) of basal-bolus insulin therapy (BBT). We aimed to develop a formula for estimating SDD and evaluate its efficacy and safety in patients with type 2 diabetes hospitalized for BBT. In the first study (n = 104), we retrospectively analyzed the relationship between peak daily dose (PDD) during hospitalization and clinical parameters. The PDD was significantly associated with fasting plasma glucose (FPG) (R = 0.449, *P* < 0.0001) and HbA1c levels (R = 0.384, *P* < 0.0001) but not body weight, body mass index, body surface area, or serum C-peptide levels. Based on the results, we developed a formula for estimating SDD using FPG levels: SDD (U/day) = 0.08 × FPG (mg/dL). In the second study (n = 405), we assessed efficacy and safety of the formula by evaluating the M-value from the daily glucose profile and assessing the frequency of hypoglycemia (blood glucose level < 70 mg/dL). When BBT was initiated using the FPG level-based formula, the M-values decreased from 61.0 ± 52.8 to 12.8 ± 10.8 (*P* < 0.0001), and hypoglycemia was observed in only 3/405 cases (0.74%) under the SDD. The FPG level-based formula is useful for estimating SDD in BBT and is safe for clinical use.

## Introduction

Insulin injection is the most effective tool for glycemic control in patients with type 1 diabetes (T1D) and type 2 diabetes (T2D). Among insulin therapies, basal-bolus insulin therapy (BBT) is widely accepted as the best option for achieving sufficient glycemic control. BBT has been proven to retard the onset and progression of microvascular complications in both insulin-dependent and non-insulin-dependent diabetes^1,2^. It is also expected that tight glycemic control by BBT in the initial stage of diabetes has the potential to prevent macrovascular complications in the future as a legacy effect^3^. Although insulin therapy is described as the final option in the recent algorithm of the American Diabetes Association (ADA)/European Association for the Study of Diabetes (EASD) for the treatment of diabetes, early introduction of insulin is recommended if there is evidence of ongoing catabolism (weight loss), if symptoms of hyperglycemia are present, or if glycated hemoglobin (HbA1c) (> 10%, 86 mmol/mol) or blood glucose levels (> 300 mg/dL) are very high^4^. Furthermore, in patients newly diagnosed with T2D, short-term BBT has been shown to reduce insulin resistance, improve β-cell function, and induce remission within several years of diagnosis^5^.

A typical starting daily dose (SDD) for BBT in patients with T1D who are metabolically stable is recommended to be determined by body weight (0.5 U/kg/day) according to the ADA/Juvenile Diabetes Research Foundation Type 1 Diabetes Sourcebook^6^. In patients with T2D, a fixed dose (10 U/day) or a body weight-based dose (0.1–0.2 U/kg/day) is recommended for initiating basal insulin therapy, although BBT is not recommended as the first insulin therapy in the ADA Standards of Medical Care in Diabetes^4^. However, BBT can be considered in patients with T2D who need to achieve good glycemic control as soon as possible, especially in those who are sick or before surgery. Thus, a formula for estimating SDD in BBT for patients with T2D is desirable.

In this study, we first retrospectively analyzed the relationship between the peak daily dose (PDD) to achieve good glycemic control and clinical parameters in 104 hospitalized patients with T2D who started insulin therapy with BBT. Based on the results, we started to use a new fasting plasma glucose (FPG) level-based formula for estimating SDD in BBT: SDD (U/day) = 0.08 × FPG (mg/dL) since January 2009 and assessed its efficacy and safety retrospectively in 405 hospitalized patients with T2D who initially received BBT for following 5 years.

## Results

### Clinical characteristics of participants in the first study

Table 1 shows the clinical characteristics of the participants upon admission in the first study. The mean HbA1c level of the participants was 10.2% ± 1.9% (93 ± 22 mmol/mol). The physicians in charge of the participants started BBT with a daily dose of 17.6 ± 4.9 U/day based on their experience. The PDD during the 13-day hospitalization was 28.4 ± 10.9 U/day. The dose was then adjusted to 26.4 ± 10.5 U/day at discharge. The M-values, representing the daily glucose profile, improved from 62.4 ± 47.8 to 12.3 ± 47.8 (*P* < 0.0001) after BBT.

**Table 1:** Clinical characteristics of the participants in the first study.

Variables	Values at admission
n (female/male patients)	104 (33/71)
Age (years)	54.0 ± 12.7
Body weight (kg)	68.3 ± 14.1
Body mass index (kg/m^2^)	25.2 ± 4.1
Body surface area (m^2^)	1.70 ± 0.20
Fasting plasma glucose (mg/dL)	198 ± 58
HbA1c [% (mmol/mol)]	10.2 ± 1.9 (93 ± 22)
Serum C-peptide (ng/mL)	2.12 ± 0.73

Continuous variables are expressed as means ± standard deviations. HbA1c, glycated hemoglobin.

### Relationships between PDD and clinical characteristics

**Figure 1:**
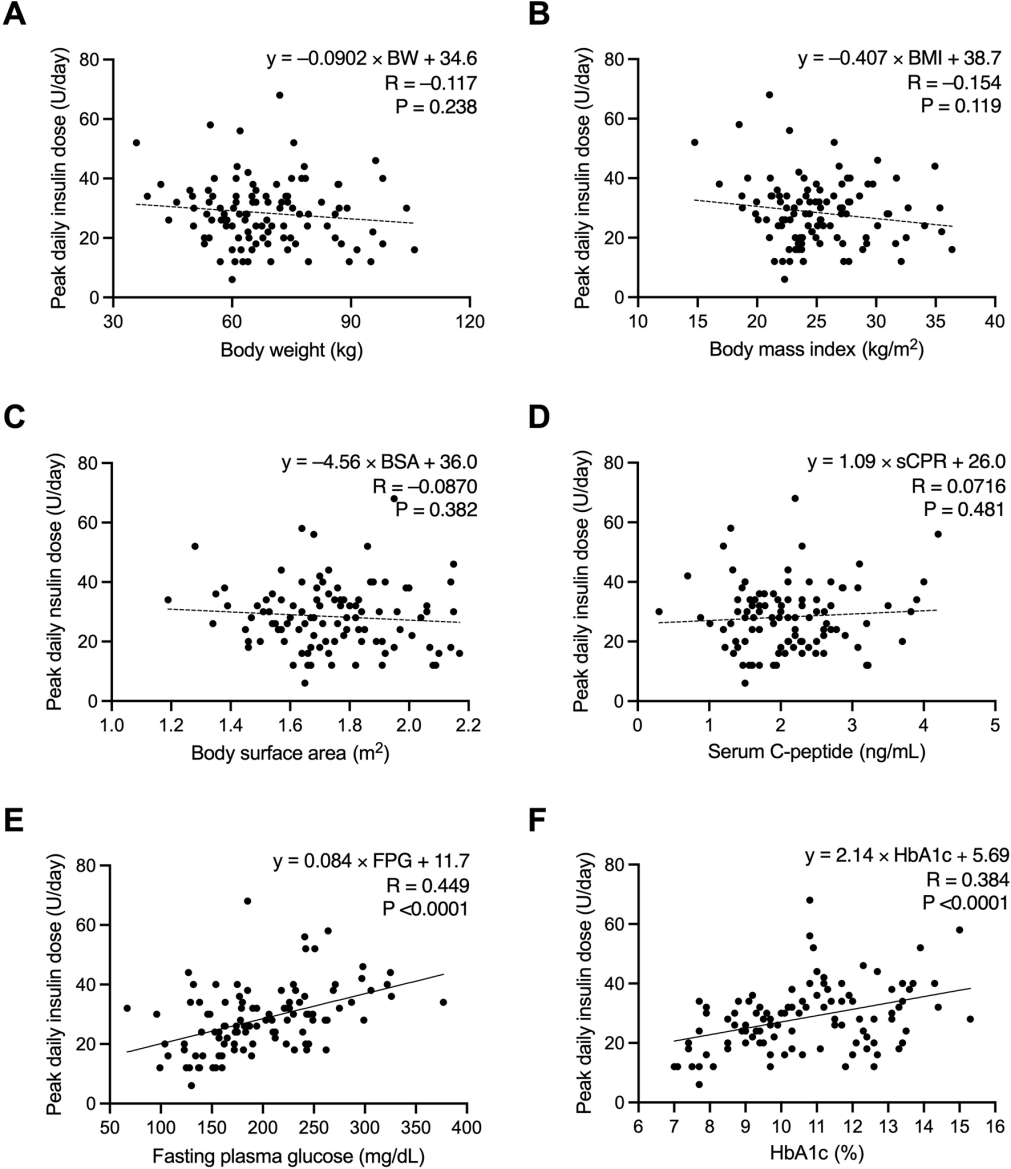
Relationships between PDD and clinical characteristics of participants on admission in the first study. The correlations between the PDD and clinical characteristics (body weight, (**A**); body mass index, (**B**) body surface area (**C**); serum C-peptide, (**D**); fasting plasma glucose, (**E**); and HbA1c [%, NGSP], (**F**) were examined using Pearson’s correlation test. The PDD is defined as the daily insulin dose that maintains fasting blood glucose levels between 100 and 130 mg/dL and postprandial blood glucose levels below 180 mg/dL. BW, body weight; BMI, body mass index; BSA, body surface area; sCPR, serum C-peptide; FPG, fasting plasma glucose; PDD, peak daily dose.

**Figure 2:**
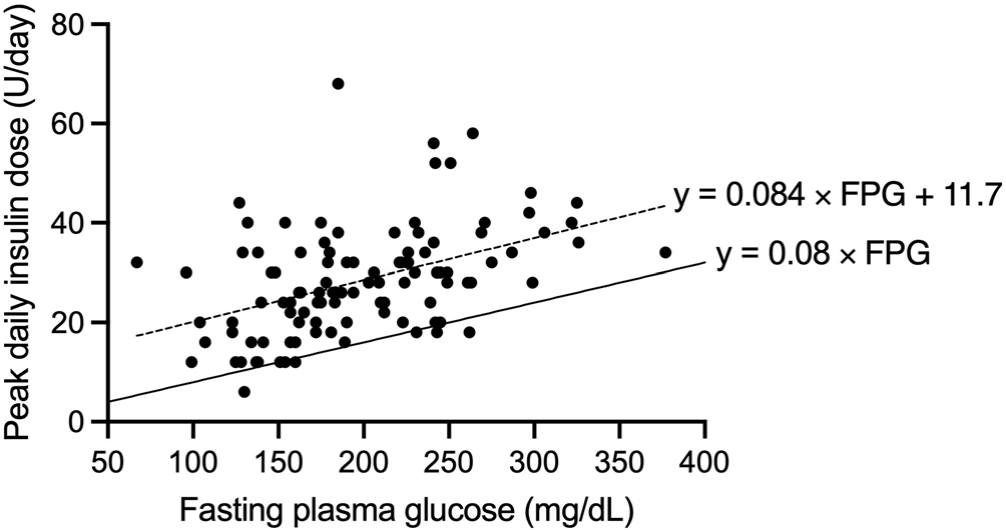
Simulation of starting daily insulin dose estimated using the FPG level-based formula. A simulation line (solid line) was drawn at “0.08 × FPG (mg/dL)” in the correlation chart between the peak daily insulin dose and FPG levels in the first study. FPG, fasting plasma glucose.

Figure 1 shows the associations between PDD and the clinical characteristics of the participants on admission. The PDD was not significantly correlated with anthropometric parameters (*i.e.*, body weight, BMI, and body surface area [BSA]) or insulin secretion capacity (serum C-peptide levels) but was correlated with glycemic parameters (*i.e.*, FPG and HbA1c levels). The correlation coefficient between PDD and FPG levels (R = 0.449) was greater than that between PDD and HbA1c levels (R = 0.384). The proportional formula between PDD and FPG levels was “PDD (U/day) = 0.084 × FPG (mg/dL) + 11.7”. When a simulation line was drawn at “0.08 × FPG (mg/dL)” in the correlation chart between the PDD and FPG levels, 98 of 104 participants (97.0%) were on or above the simulation line (Fig. 2). Therefore, we proposed the following formula for estimating SDD in BBT: SDD (U/day) = 0.08 × FPG (mg/dL).

### Efficacy and safety of FPG level*-*based formula for SDD in BBT

Thereafter, we started to use the FPG level-based formula for estimating SDD since January 2009 and assessed its efficacy and safety for following 5 years. Table 2 shows the clinical characteristics of the participants upon admission in the second study. During the study period, the physicians in charge of the participants started BBT with the SDD determined using the FPG level-based formula. We therefore retrospectively collected more detailed information on the participants’ clinical characteristics, including renal function (estimated glomerular filtration rate [eGFR]), diabetic microvascular complications, and prehospital treatment for T2D, to clarify the characteristics associated with hypoglycemia under BBT, starting with the dose calculated using the FPG level-based formula. BBT was started with a daily dose of 14.2 ± 4.5 U/day, which was increased to 25.6 ± 12.2 U/day to achieve the glycemic goal and adjusted to 22.9 ± 13.4 U/day at discharge. BBT was initiated with three daily bolus injections in 208 cases (51%) and was changed to three daily bolus and one daily basal (four times) injections in 117 cases. Accordingly, 91 (22%) and 314 patients (78%) were injected with insulin analogs three and four times a day at discharge, respectively. Through BBT, glycemic control improved as follows: the FPG level decreased from 181 ± 56 to 121 ± 24 mg/dL (*P* < 0.0001); the postprandial plasma glucose level 2 h after breakfast decreased from 303 ± 83 to 179 ± 48 mg/dL (*P* < 0.0001); and the M-values decreased from 61.0 ± 52.8 to 12.8 ± 10.8 (*P* < 0.0001). Hypoglycemia (blood glucose level < 70 mg/dL) was recorded under the SDD in three out of 405 cases (0.74%). The clinical characteristics of the patients with hypoglycemia are shown in Table 3. One patient experienced hypoglycemic symptoms (cold sweats), and two patients were diabetic drug-naïve. The eGFR was preserved (≥ 60 mL/min/1.73 m^2^) in all three patients.

**Table 2:** Clinical characteristics of participants in the second study.

Variables	Values at admission
n (female/male patients)	405 (119/286)
Age (years)	60.0 ± 12.0
Body weight (kg)	66.6 ± 14.4
Body mass index (kg/m^2^)	24.8 ± 4.4
Body surface area (m^2^)	1.71 ± 0.21
Fasting plasma glucose (mg/dL)	181 ± 56
HbA1c [% (mmol/mol)]	9.9 ± 2.2 (85 ± 24)
Serum C-peptide (ng/mL)	2.03 ± 0.96
Estimated glomerular filtration rate (mL/min/1.73 m^2^)	77.1 ± 28.2
Microvascular complications	
Neuropathy [n (%)]	137 (34)
Retinopathy [n (%)]	85 (21)
Nephropathy [n (%)]	126 (31)
Prehospital medication	
SU [n (%)]	137 (34)
Oral hypoglycemic agent other than SU [n (%)]	80 (20)

Continuous variables are expressed as mean ± standard deviation. HbA1c, glycated hemoglobin; SU, sulfonylurea.

**Table 3:** Clinical characteristics of patients with hypoglycemia under SDD in the second study.

Case	BG*(mg/dL)	Symptoms	Age (years)	Sex	T2D duration (years)	Prehospital treatment	HbA1c [% (mmol/mol)]	FPG (mg/dL)	SDD (U)	eGFR (mL/min)
1	54	Yes	68	M	10	SU	7.5 (58)	92	6	73.6
2	68	No	70	F	< 1	None	14.1 (130)	157	12	75.0
3	69	No	55	F	< 1	None	11.0 (97)	256	20	109

*BG level indicating hypoglycemia.

BG, blood glucose level; eGFR, estimated glomerular filtration rate; F, female; FPG, fasting plasma glucose; HbA1c, glycated hemoglobin; M, male; SDD, starting daily dose; SU, sulfonylurea.

## Discussion

In this study, we showed that the PDD for achieving good glycemic control was significantly associated with glycemic parameters (*i.e.*, FPG and HbA1c levels) among hospitalized patients with T2D who initially received BBT. Surprisingly, the PDD was not correlated with anthropometric parameters including body weight and insulin secretion capacity in these patients. Accordingly, we proposed the following new FPG level-based formula for estimating SDD in BBT: SDD (U/day) = 0.08 × FPG (mg/dL). Indeed, patients with T2D who started BBT with the daily dose calculated using this formula achieved better glycemic control with a low incidence of hypoglycemia.

According to the clinical guidelines for diabetes treatment, the SDD in BBT has been estimated using body weight in patients with T1D: SDD (U/day) = 0.5 × body weight (kg)^6^. A similar formula has also been suggested for estimating SDD in BBT for patients with T2D, according to the diabetes treatment guidelines issued by the Japan Diabetes Society (JDS): SDD (U/day) = 0.1–0.2 × body weight (kg)^7^. The body weight-based formula for estimating SDD has been used since the first patient received an insulin injection in 1922^8^. Most studies in patients with insulin-deficient T1D receiving continuous insulin infusion have suggested that the total daily dose (TDD) should be approximately 0.5–0.6 U/kg regardless of ethnic background^9^. Therefore, the body weight-based formula for estimating SDD is considered reasonable for patients with T1D. In contrast, to the best of our knowledge, only a few studies have assessed the factors associated with TDD in patients with T2D. A retrospective study was performed to identify the factors associated with TDD in Chinese patients with T2D who received BBT for glycemic control during hospitalization^10^. The study reported that the TDD was positively associated with glycemic parameters, including HbA1c, fasting blood glucose (FBG), and postprandial blood glucose levels, as well as BMI upon admission. Based on these findings, T2D patients with higher blood glucose levels are considered to have a greater demand for exogenous insulin because hyperglycemia per se reflects lower insulin secretion in addition to insulin resistance. These findings might also be affected by the characteristics of East Asian patients with T2D, among whom β-cell dysfunction rather than insulin resistance can be the primary cause of hyperglycemia^11^. However, in our first study, the PDD did not correlate with serum C-peptide levels. Furthermore, although a significant association was detected between PDD and body weight (R = 0.135, *P* = 0.0066) in the second study with four times the number of participants that were presented in the first study, the correlation coefficient was significantly lower than that between PDD and FPG levels (R = 0.589, *P* < 0.0001). Therefore, we considered it appropriate to use FPG levels instead of serum C-peptide levels or body weight to estimate SDD, at least in East Asian patients with T2D who start BBT.

Based on the proportional formula between PDD and FPG levels in patients with T2D, we proposed the FPG level-based formula for estimating SDD in BBT. When the body weight-based formula “SDD (U/day) = 0.2 × body weight (kg)” was applied to the participants in the second study with a mean body weight of 66.6 kg, SDD was estimated to be approximately 13 U/day. This dose is similar to the SDD calculated using the FPG level-based formula where BBT was started at a dose of 14.2 ± 4.5 U/day in the second study. However, as the PDD was not correlated with body weight in the first study, the body weight-based formula could underestimate SDD, particularly in T2D patients with a lower body weight and higher blood glucose level. Such patients are expected to achieve good glycemic control in a shorter period by starting BBT at the SDD estimated using the FPG level-based formula.

There is a concern that a part of preceding oral hypoglycemic agents and GLP-1 receptor agonists, *e.g.*, sulfonylureas, once-weekly dipeptidyl peptidase-4 (DPP-4) inhibitors, and long-acting GLP-1 receptor agonists, affected FPG levels before starting BBT. In the second study, 137 participants had been treated with sulfonylureas and one participant with a GLP-1 receptor agonist, liraglutide. The participants treated with sulfonylureas (n = 137) showed lower FPG levels (159 ± 5 vs. 191 ± 3 mg/dL, *P* < 0.0001) and consequently started BBT with a lower SDD (12.8 ± 0.4 vs*.* 15.3 ± 0.3 U/day, *P* < 0.0001) as compared to those without (n = 268). However, PDD in those with sulfonylureas was significantly higher than PDD in those without (27.9 ± 1.0 vs*.* 24.5 ± 0.7 U/day, *P* = 0.0072). These data suggest that the FPG level-based formula could underestimate SDD, particularly in T2D patients treated with anti-diabetic drugs with a long duration of action. In a future study to develop more accurate FPG level-based formula for estimating SDD, the anti-diabetic drugs should be withdrawn prior to a period longer than the drug action time (*e.g.*, 12–24 h for sulfonylureas) to mitigate their pharmacological effects on FPG levels.

Notably, two of the three patients who experienced hypoglycemia under the SDD in the second study were drug-naïve with a diabetes duration of less than 1 year. Drug-naïve patients are generally expected to have rapid improvements in β-cell function following BBT after recovery from glucotoxicity. Another patient with hypoglycemia was treated with sulfonylurea. This patient was also expected to have improvements in β-cell function after recovery from secondary failure of sulfonylurea therapy. Therefore, we should consider earlier insulin dose titration in such patient groups when BBT is introduced with the SDD estimated using the FPG level-based formula.

Our study had several limitations. First, because this was a single-arm retrospective study without a control group, we could not show the advantages of the FPG level-based formula compared to the traditional body weight-based formula. Therefore, we plan to conduct a prospective study to compare the two formulas in patients with T2D who begin BBT. Second, the study was performed during a 2-week hospitalization period, and the availability and safety of the FPG level-based formula should be assessed in a long-term outpatient setting. Third, BBT was initiated after cessation of oral hypoglycemic agents and GLP-1 receptor agonists. In the recent ADA/EASD algorithm for the treatment of diabetes^4^, metformin is recommended to be continued as long as it is tolerated and not contraindicated. As insulin therapy is recommended to be accompanied with metformin, another study should be performed to estimate SDD in BBT combined with metformin administration.

In summary, the PDD for achieving good glycemic control was strongly associated with FPG levels before starting BBT in patients with T2D who received insulin therapy. Therefore, we proposed the following FPG level-based formula for estimating SDD in BBT: SDD (U/day) = 0.08 × FPG (mg/dL). When BBT was started from a daily dose calculated using the FPG level-based formula, glycemic control improved significantly during 2 weeks of hospitalization, and hypoglycemia was observed in only 0.74% of the patients under the SDD. Therefore, we conclude that the FPG level-based formula can be a good option for estimating SDD in BBT for patients with T2D requiring rapid glycemic control.

## Methods

### Participants

Patients with T2D who were admitted to our hospital to start insulin therapy for glycemic control were enrolled in the first study during January 2006–December 2008 (n = 104) and the second study during January 2009–December 2014 (n = 405). The participants were introduced to insulin therapy using BBT owing to poor glycemic control with a HbA1c level > 8% (64 mmol/mol) or with ongoing catabolism (weight loss) and/or symptoms of hyperglycemia, such as thirst, polydipsia, and polyuria. The BBT initiation program at our hospital was scheduled for 13 days. Patients with an uncontrolled endocrine disease, infection, malignant tumor under treatment, or ketoacidosis and those receiving steroid therapy and hemodialysis were excluded. The study protocols were approved by the Nippon Medical School Hospital Ethics Committee, and they conformed to the provisions of the Declaration of Helsinki in 1995 (revised in Edinburgh, 2000). All patients provided informed consent before enrollment. All treatments were provided as part of the routine care.

### Clinical measurements

All participants underwent physical examinations, including height and body weight assessments, on the morning after admission. BSA was calculated using the Du Bois formula according to height and body weight^12^. Blood samples were collected after an overnight fast on the second day of admission and on the second or third day before discharge. Plasma glucose levels were measured by the glucose oxidase method (ADAMS Glucose GA-1170; Arkray, Kyoto, Japan). The daily glucose profile was determined by measuring plasma glucose levels at seven time points (30 min before and 2 h after each meal and at bedtime). The M-value was calculated using the Schlichtkrull formula^13^. HbA1c levels were measured using high-performance liquid chromatography (ADAMS A1c HA-8160; Arkray) and expressed as the percentage of the National Glycohemoglobin Standardization Program (NGSP) according to the JDS guideline^14^. If necessary, the NGSP values (%) were converted to the International Federation of Clinical Chemistry (IFCC) values (mmol/mol) using the following formula: IFCC = (10.93 × NGSP) − 23.50. Serum C-peptide levels were measured using a chemiluminescent enzyme immunoassay (Fujirebio Inc., Tokyo, Japan). The eGFR was calculated using the following formula: eGFR (mL/min/1.73 m^2^) = 194 × Cr^−1.094^ × age^−0.287^ (× 0.742 for women)^15^.

### Assessment of microvascular complications

Neuropathy was screened using the criteria for typical diabetic peripheral neuropathy proposed by the ADA^16^. The presence of typical diabetic peripheral neuropathic symptoms (decreased sensation and positive neuropathic sensory symptoms such as numbness, prickling or stabbing, burning, or aching pain) in the lower legs or an abnormal (decreased or absent) Achilles tendon reflex was diagnosed as neuropathy (including possible neuropathy). Retinopathy was diagnosed by an ophthalmologist using ophthalmoscopy according to the Davis criteria^17^. Nephropathy was diagnosed based on the presence of albuminuria (urinary albumin excretion level ≥ 30 mg/g･Cr [spot]) or an eGFR of < 30 mL/min/1.73 m^2^ ^18^.

### Medication and insulin therapy

If the participants were treated with oral hypoglycemic agents or glucagon-like peptide 1 (GLP-1) receptor agonists on admission, the drugs were withdrawn on the second day of admission. BBT was introduced for all participants in both studies, and the SDD was determined using the following formula during the second study term: SDD (U/day) = 0.08 × FPG (mg/dL). In this study, actual SDD was modified to within 2 U/day from the calculated SDD. Thereafter, the actual SDD was divided into three or four daily insulin injections: three bolus injections of ultrarapid insulin analog (aspart, glulisine, or lispro) before meals for all participants and one bedtime basal injection of insulin glargine or detemir for participants with FPG levels ≥ 150 mg/dL. The dose of insulin injection was adjusted by physicians to within 4 U for each injection. The goals of glycemic control were to maintain FBG levels between 100 and 130 mg/dL and postprandial blood glucose (PBG) levels below 180 mg/dL according to the recommendations of the JDS^18^.

### Diet therapy

During hospitalization, dietary energy intake (kcal/day) was restricted to 27.5 kcal/kg of ideal body weight (IBW), according to the recommendations of the JDS^18^. IBW was calculated using the following formula because a body mass index (BMI) of 22 kg/m^2^ is regarded as ideal for adult Japanese individuals: IBW (kg) = [height (m)]^2^ × 22 (BMI, kg/m^2^). Daily dietary energy intake was divided approximately equally between breakfast (08:00 h), lunch (12:00 h), and dinner (18:00 h). Each diet contained approximately 20–25% of energy as fat, 15–20% as protein, and 55–60% as carbohydrate. To evaluate the efficacy of diet therapy, we asked participants to maintain their physical activity within their usual intensity without a specific exercise program.

### Statistical analysis

Continuous variables are described as mean ± standard deviation or median and interquartile range. Categorical variables are expressed as numbers and percentages. Differences in variables between admission and discharge were analyzed using a paired t-test. Correlations between the maximum insulin dose and other continuous variables were examined using Pearson’s correlation test. Statistical significance was set at *P* < 0.05. All analyses were performed using the JMP software (version Pro 16.0; SAS Institute Inc., Cary, NC, USA).

## Data Availability

All data generated or analyzed during this study are included in this published article.
